# Validity and reliability of remote administration of the one-leg stand, timed up and go and 30-second sit-to-stand via video call in patients with lumbar spinal stenosis awaiting decompression surgery

**DOI:** 10.1186/s12891-026-10149-9

**Published:** 2026-07-08

**Authors:** Rikard Hanafi, Christian Ernest, Emelie Karlsson, Mike K. Kemani, Jo Nijs, Mari Lundberg, Max Jakobsson

**Affiliations:** 1 Back in Motion Research Group, Department of Health Promoting Science, Sophiahemmet University, Stockholm, 114 86 Sweden; 2 Pain in Motion Research Group, Department of Physiotherapy, Human Physiology and Anatomy, Faculty of Physical Education & Physiotherapy, Vrije Universiteit, Brussel, 1050 Belgium; 3 Medical Unit Allied Health Professionals, Karolinska University Hospital, Theme Women’s Health and Allied Health Professionals, Solna, 171 76 Sweden; 4 Capio Spine Center Göteborg, Västra Frölunda, Gothenburg, 421 30 Sweden; 5 Department of Clinical Neuroscience, Karolinska Institutet, Stockholm, 171 77 Sweden; 6 PijnPraxis.be Interdisciplinary Practice for Pain Management, Leopoldsburg, 3970 Belgium; 7 Sahlgrenska Academy, GPCC. University of Gothenburg Centre for Person-Centred Care (GPCC), University of Gothenburg, Gothenburg, 405 30 Sweden; 8 Division of Physiotherapy, Department of Health and Rehabilitation, Institute of Neuroscience and Physiology, Sahlgrenska Academy, University of Gothenburg, Gothenburg, 405 30 Sweden

**Keywords:** Lumbar spinal stenosis, physical capacity tests, performance-based physical function tests, Telehealth, Remote assessment, Videoconferencing, Validity, Reliability

## Abstract

**Background:**

Lumbar spinal stenosis commonly affects walking ability and balance in older adults. Physical capacity tests provide valuable objective measures beyond patient-reported outcomes, but time and travel barriers hinder access to in-person testing. Remote video-based testing could improve accessibility, although evidence of its validity and reliability in this patient group remains limited. This study aimed to evaluate the validity and reliability of three commonly used tests administered remotely via video calls, compared with in-person assessments in a clinical setting.

**Methods:**

Thirty-four patients scheduled for decompression surgery were enrolled from a Swedish spine clinic, of whom 28 completed at least one test session. Participants performed the one-leg stand, timed up and go, and 30-second sit-to-stand tests. Two consecutive trials of the full test battery were performed in two settings: remotely via video call from patients’ homes and in person at the clinic. Participants were randomised to the order of test settings. Remote sessions were recorded and independently rated. Criterion validity (comparison between remote and in-person assessments) and intra- and inter-rater reliability were assessed using intraclass correlation coefficients, with values ≥ 0.70 considered sufficient for both criterion validity and reliability. Bland–Altman limits of agreement, standard error of measurement, and smallest detectable change were also calculated.

**Results:**

Intraclass correlation coefficients for criterion validity ranged from 0.83 to 0.88. Intra-rater reliability ranged from 0.95 to 0.97 in person and 0.80 to 0.98 remotely. Inter-rater reliability was 0.98 for all three tests. For the one-leg stand, confidence intervals were wider and measurement errors were greater than for the other tests. The median interval between sessions was three days. No adverse events occurred during testing.

**Conclusions:**

Remote video-based assessments of the one-leg stand, timed up and go, and 30-second sit-to-stand tests demonstrated sufficient criterion validity and intra- and inter-rater reliability in patients with lumbar spinal stenosis awaiting decompression surgery. However, the one-leg stand showed greater measurement variability and should be interpreted with caution. Taken together, these findings suggest that remote assessment of these tests may serve as a valid and reliable complement to in-person evaluation. Larger studies are needed to assess measurement error and responsiveness.

## Background

 Lumbar spinal stenosis (LSS) is a common degenerative condition in older adults associated with considerable pain and functional impairment [1, 2]. According to clinical diagnostic criteria, a meta-analysis estimated prevalence rates of about 11% in the general population and 25–39% in clinical populations [3]. The prevalence of LSS increases with age and remains the primary indication for spine surgery among older adults [4].

LSS is characterised by neurogenic claudication, typically presenting as leg pain, numbness, or weakness triggered by walking and standing. These symptoms frequently impair walking ability and balance, thereby limiting participation in daily activities [1]. Patients with persistent, functionally limiting symptoms despite conservative treatment may be referred for decompression surgery to relieve neural compression, with the primary aim of reducing symptoms and improving physical function [4, 5].

Outcome evaluation after surgery and/or rehabilitation in LSS primarily relies on patient-reported outcome measures (PROMs) [6, 7]. While PROMs are essential for capturing patients’ perceived function and symptom experience, they show modest correlations with standardised objective measures of function in LSS [8–10]. Standardised performance-based tests can therefore provide complementary, clinically relevant information on balance, mobility, and lower-body strength, supporting comprehensive assessment and informing clinical decision-making in LSS [9, 11]. Accordingly, recent international Delphi consensus recommendations for LSS emphasise that outcome assessment should include objective, performance-based measures of function, particularly for evaluating observed walking capacity and one-leg balance, complementing relevant PROMs [12].

A performance-based physical capacity test involves the patient performing a standardised activity, administered by a rater according to predefined criteria such as test timing or repetition counts [13]. Some standardised tests require specialised equipment, such as a treadmill, which necessitates in-clinic visits [14, 15]. Other simpler tests, commonly used in LSS to evaluate static and dynamic balance, functional mobility, and lower-body strength, include the one-leg stand (OLS), timed up and go (TUG), and 30-second sit-to-stand (30-s STS) [9, 11, 16–18]. The TUG has demonstrated acceptable validity, reliability, and responsiveness in lumbar spine surgery populations, including patients with LSS [19]. Evidence for the 30-s STS and OLS specifically in patients with LSS is limited, but both tests have demonstrated acceptable measurement properties in related populations, including older adults and patients with low back pain [20–22].

Given their simplicity and minimal resource requirements, such tests have potential for remote assessment via video call in patients’ homes. However, in-person assessment remains the clinical standard for administering common physical capacity tests, yet accessibility is often limited by time- and transport-related barriers [23]. Telehealth has been shown to reduce these barriers and improve access to musculoskeletal care [24]. Despite increasing interest in telehealth for assessing physical function, the evidence base for remote physical capacity testing in musculoskeletal conditions remains limited.

This is reflected in a recent systematic review by Walsh et al. [25], which reported low- to very-low-quality evidence regarding the measurement properties of performance-based physical tests administered via telehealth across chronic conditions. This included limited evidence for criterion validity, defined as the correlation between telehealth and in-person administration of the same test, with in-person administration considered the reference standard. The authors acknowledged the lack of studies in chronic musculoskeletal populations and highlighted the need for future research examining the measurement properties of performance-based physical function tests administered via telehealth in this population.

Our research team has recently implemented the OLS, TUG, and 30-s STS as part of a comprehensive outcome battery in an ongoing randomised controlled trial evaluating the effects of an early digital rehabilitation programme for LSS patients undergoing decompression surgery [26]. In this trial, these tests are administered remotely via a secure videoconferencing platform. Preliminary feasibility results demonstrated that these remote procedures are safe and well received by both patients and raters [27]. However, beyond feasibility and safety, acceptable measurement properties must be established in the specific context of use [28]. Given the potential of home-based testing to improve accessibility and support equitable, person-centred care, clinical implementation requires evidence of adequate measurement properties in the target population. Specifically, the criterion validity and reliability of these tests when performed at home and assessed remotely via video calls have not yet been established in patients with LSS awaiting decompression surgery.

## Methods

### Aims

The aim of this study was to evaluate the criterion validity and intra- and inter-rater reliability of remote video-based assessments of the one-leg stand (OLS), timed up and go (TUG), and 30-second sit-to-stand (30-s STS) in patients with LSS awaiting decompression surgery.

### Study design

A repeated-measures design was employed to evaluate criterion validity and reliability. Measurement property definitions and terminology adhered to the COnsensus-based Standards for the selection of health Measurement INstruments (COSMIN) [29].

### Participants and setting

**Fig. 1:**
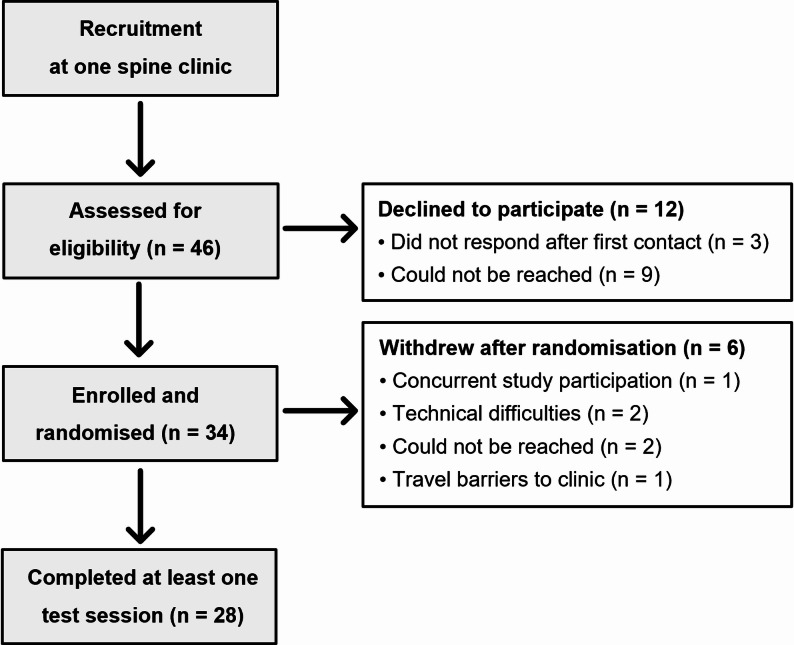
Participant recruitment flowchart

Patients with LSS were consecutively recruited from a single spine clinic in Gothenburg, Sweden (Fig. 1). A study-associated physiotherapist screened patients for eligibility against the following inclusion criteria: (1) aged ≥ 18 years; (2) diagnosed with central LSS and scheduled for decompression surgery; (3) proficient in Swedish; (4) access to a computer, tablet, or smartphone with a stable internet connection at home; and (5) able to use BankID (a national electronic identification system in Sweden) for secure authentication. Patients with medical conditions that precluded safe participation (e.g., untreated or unstable cardiovascular disease) were excluded.

### Sample size

We aimed to include at least 25 participants to estimate an intraclass correlation coefficient (ICC) of 0.70 with a 95% confidence interval of ±0.20, based on two repeated measurements per participant, using the formula proposed by Giraudeau and Mary [30], as presented by de Vet et al. [28] (Eq. 1).

1 $$\:\mathrm{n}\:=\:\frac{8\:{\mathrm{z}}_{1-{\upalpha\:}/2}^{2}{\left(1-\mathrm{I}\mathrm{C}\mathrm{C}\right)}^{2}{\left[1+\left(\mathrm{m}-1\right)\mathrm{I}\mathrm{C}\mathrm{C}\right]}^{2}}{m\left(m-1\right){w}^{2}}$$

Formula for calculating the sample size for an intraclass correlation coefficient (ICC), as proposed by Giraudeau and Mary [30]. In the formula, m represents the number of measurements per participant, w denotes the total width of the 95% confidence interval (CI) for the ICC, and z is the standard normal deviate corresponding to the chosen confidence level (1.96 for 95%).

### Participant characteristics

Demographic data, including age, sex, height, weight, educational level, employment status, prior surgery, and pain duration, were collected using questions from the Swedish National Spine Register (SweSpine) baseline questionnaire [31]. Body mass index (BMI) was calculated from self-reported height and weight. Disability related to LSS was assessed using the Oswestry Disability Index (ODI), which ranges from 0 to 100, with higher scores indicating greater disability [32]. Pain intensity was assessed using the Numeric Pain Rating Scale (NPRS), which ranges from 0 (no pain) to 10 (worst imaginable pain), and was reported separately for leg and back pain [33]. Participants also reported use of walking aids, current analgesic use, and comorbidities perceived to affect walking ability, balance, or leg strength.

### Test battery

#### One-Leg Stand (OLS), eyes open

Postural balance was assessed using the OLS as described by Maribo et al. [20]. Participants stood on one leg, eyes open and unassisted, for as long as possible (up to 60 s). The rater began timing when the opposite foot left the ground and stopped when it touched the ground again. Participants were instructed to lift the foot approximately 10 cm and place it behind the weight-bearing leg. In each trial, participants performed two attempts per leg. The trial score was based on the best attempt for each leg, with the final trial score being the shorter of the two best-per-leg times (“worst leg”) [20].

#### Timed Up and Go (TUG)

Functional mobility and dynamic balance were assessed using the TUG [34]. Participants were timed as they stood from a chair without armrests, walked 3 m at a comfortable pace, turned 180 degrees, walked back, and sat down. During in-person testing, a chair with a seat height of 45 cm was used. For remote testing, participants selected a preferred chair with a seat height of 45–47 cm. Before the test began, each participant was instructed to sit with their back against the backrest. Participants could use a mobility aid if needed. Each trial consisted of a single timed attempt, and the time was recorded in seconds.

#### 30-Second Sit-To-Stand (30-s STS)

Functional leg strength was assessed using the 30-s STS [35]. Participants were instructed to sit on a chair against a wall, with arms crossed over their chest and one foot slightly in front of the other. From this position, they were instructed to complete as many full sit-to-stand cycles as possible in 30 s. A full repetition was defined as standing fully upright, then sitting back down with the buttocks touching the chair, while keeping the arms crossed throughout. Each trial consisted of a single 30-second period, and the trial score was the number of full sit-to-stand repetitions.

### Test procedures

**Fig. 2:**
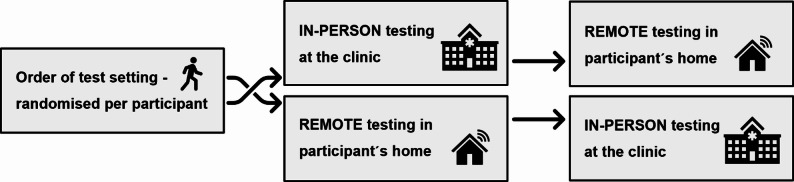
Schematic overview of the randomised test order

Participants completed one test session remotely via a secure healthcare videoconferencing platform and another session in person at the recruiting clinic. The order of test settings (remote or in-person first) was randomised using a predefined block randomisation scheme (blocks of six, generated via Random.org) and concealed within opaque, pre-numbered envelopes (Fig. 2).

Before the remote session, participants received written instructions on how to use the videoconferencing platform and perform the three tests. To mirror the in-clinic testing environment, participants were instructed to use a clear, level floor area with sufficient space to perform the tests safely. A three-metre string and tape were provided to mark the required walking distance for the TUG. Participants were instructed to position the camera so that the full body was visible throughout all tests.

Before initiating testing in the remote setting, the raters administered a brief safety screening, based on a previous study with a similar setup, to ensure safe participation [36]. This comprised five questions covering: the presence of another person at home, appropriate footwear, recent falls, dizziness or diaphoresis, and new-onset chest pain. In addition, the raters were instructed to visually confirm that the testing area was suitable, with sufficient space and a clear, level, firm floor surface.

In both settings, participants completed two consecutive trials of the full test battery. Tests were performed in a fixed order (OLS → TUG → 30-s STS). Before each trial, participants rated their current pain intensity using the NPRS [37]. After each test, participants rated perceived exertion using the Borg scale [38], with short rest periods between tests as needed for recovery. In addition, raters were instructed to monitor and document any adverse events during testing.

### Evaluation of validity and reliability

Two raters administered the assessments in both settings, with each rater evaluating the same participants in each setting. All remote sessions were screen-recorded and later independently evaluated by a third rater, who was blinded to the original raters’ scores. All three raters were licensed physiotherapists with experience of the study population. They followed a standardised assessment protocol and used a standard stopwatch for all tests.

**Fig. 3:**
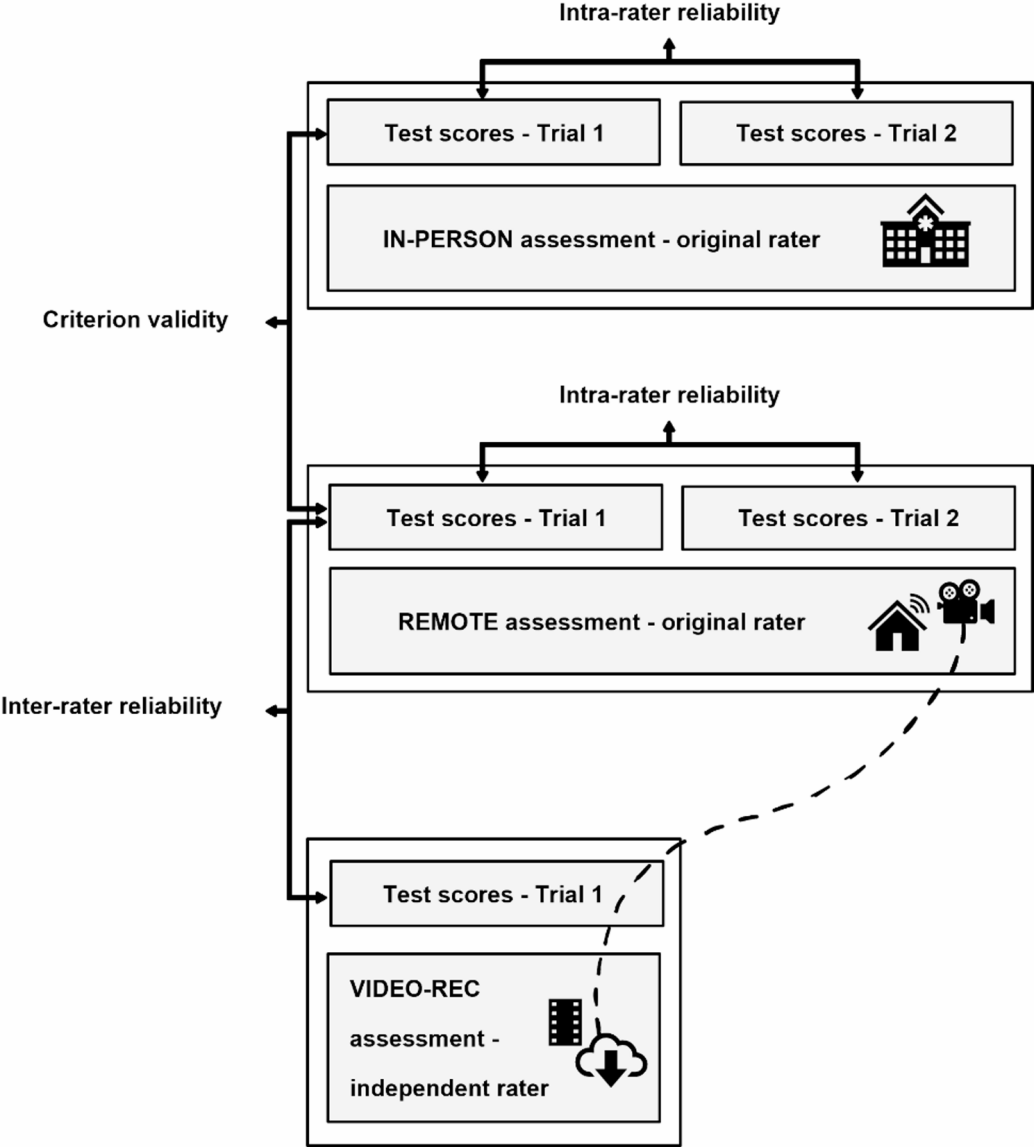
Overview of the assessments’ relationships to the evaluated measurement

Criterion validity was assessed by examining the association between remote and in-person test scores, with in-person performance considered the reference standard (Fig. 3), consistent with the COSMIN-based approach described by Walsh et al. [25]. To reflect typical clinical practice, only scores from the first trial were included in the primary analysis.

Intra-rater reliability was assessed by comparing each original rater’s trial 1 and trial 2 scores, separately for the in-person and remote settings. Inter-rater reliability was assessed by comparing the original raters’ test scores from the remote assessment (trial 1) with the independent rater’s scores based on the video recording of the same trial (Fig. 3). When scoring the video recordings, the independent rater could observe the original rater’s interaction with the participant and the initiation of the tests but could not see or hear when the original rater stopped timing or view the recorded test results.

### Statistical analysis

Statistical analyses were conducted using IBM SPSS Statistics for Windows, version 29.0 (IBM Corp., Armonk, NY, USA), and Microsoft 365 Excel (Microsoft Corporation, Redmond, WA, USA). Descriptive statistics summarised baseline demographics and clinical characteristics. Continuous variables were reported as mean (standard deviation, SD) if approximately normally distributed, and as median (interquartile range, IQR) otherwise. Normality was assessed by visual inspection of histograms and Q–Q plots. Categorical variables were summarised as counts and percentages.

Criterion validity and inter-rater reliability were assessed using ICC(2,1), a two-way random-effects model with absolute agreement (single measures) and 95% CIs, allowing generalisation beyond the specific raters. Intra-rater reliability was assessed using ICC(3,1), a two-way mixed-effects model with absolute agreement (single measures) and 95% CIs, treating the rater as a fixed effect [39]. According to the COSMIN criteria for sufficient criterion validity and reliability, ICC values ≥ 0.70 were considered sufficient for group-level comparisons [28, 40]. ICC estimates were further interpreted using the categories proposed by Koo and Li [39] (< 0.50 poor, 0.50–0.75 moderate, 0.75–0.90 good, and > 0.90 excellent), taking their 95% CIs into account.

We also evaluated measurement error by estimating the limits of agreement (LoA), the standard error of measurement (SEM), and the smallest detectable change at the 95% confidence level (SDC95), in accordance with COSMIN [40]. Bland–Altman analyses were used to estimate the mean difference and 95% limits of agreement, calculated as the mean difference ± 1.96 × SD of the differences between paired test scores, and to explore potential systematic or proportional bias across the measurement range [41]. The standard error of measurement (SEM) was calculated as SD × √(1 − ICC), where SD is the pooled standard deviation of the paired test scores [24]. The smallest detectable change at the 95% confidence level (SDC95) was calculated as 1.96 × √2 × SEM, representing the smallest change that exceeds measurement error [28, 42].

## Results

Thirty-four participants were randomised (Fig. 1). Six participants dropped out after randomisation but before any test session was scheduled. Baseline characteristics for the remaining 28 participants are presented in Table 1. Complete paired data were available for 22–28 participants, depending on the specific test and analysis. Missing data resulted from three sources: one participant missed an entire remote session for personal reasons; individual tests were missing because participants opted out due to increased pain during testing; and, for the inter-rater reliability analysis, additional data were lost due to a technical failure of the screen recording software.

**Table 1:** Participant characteristics *N* = 28

Age (years), mean (SD)	69 (9.2)
Sex	
Male, *n* (%)	10 (36%)
Female, *n* (%)	18 (64%)
BMI (kg/m²), mean (SD)	28.1 (4.1)
Educational level	
Primary school, *n* (%)	4 (14%)
High school, *n* (%)	10 (36%)
University, *n* (%)	14 (50%)
Employment status	
Working, *n* (%)	9 (32%)
Retired, *n* (%)	18 (64%)
Unemployed, *n* (%)	1 (4%)
ODI (0–100), mean (SD)	36 (15.2)
Use of walking aid, *n* (%)	9 (32%)
Comorbidity affecting walking, balance, or leg strength, *n* (%)	12 (43%)
Pain intensity, past week (NPRS 0–10)	
Leg pain, mean (SD)	5.8 (2.3)
Back pain, mean (SD)	5.2 (2.2)
Analgesic use, *n* (%)	20 (71%)
Prior lumbar spine surgery, *n* (%)	9 (32%)

*SD*  Standard deviation, *n*  Number, *%* Percent, *BMI*  Body mass index, *ODI*  Oswestry Disability Index 2.0, *NPRS*  Numeric Pain Rating Scale

Participants had a mean age of 69 years (SD 9.2), with 64% (*n* = 18) being female. Half had university-level education (*n* = 14), and 64% (*n* = 18) were retired. The mean ODI score was 36 (SD 15.2), indicating moderate disability [32]. Participants reported a mean leg pain rating of 5.8 (SD 2.3) and back pain of 5.2 (SD 2.2), both reflecting moderate pain intensity [43].

The median time between testing sessions was three days (IQR 1–6). Pre-test pain ratings were 3.64 (SD 2.08) in the in-person setting and 3.73 (SD 2.39) in the remote setting.

Twenty-five participants (89%) responded to the preference and safety questions regarding the perceived differences between testing settings. Regarding patient preferences, eight participants (32%) favoured digital testing, eight (32%) preferred in-person assessments, and nine (36%) reported no preference between the two formats. Furthermore, 24 participants (96%) reported no perceived difference in safety between remote and in-person testing, while one participant preferred the in-person setting. No adverse events occurred during testing.

### Criterion validity

For criterion validity, ICC estimates were above the predefined threshold of 0.70 for all three tests, though the lower bound of the 95% CI for OLS fell below this threshold (0.64) (Table 2). Four participants (16%) reached the maximum OLS test time (60 s). SEM and SDC95 values are reported in Table 2.

**Table 2:** Criterion validity for remote compared with in-person assessment

Test	*n*	Remote Mean (SD)	In-person Mean (SD)	ICC(2,1) [95% CI]	SEM	SDC95
OLS (s)*	25	18.53 (20.32)	23.19 (21.47)	0.83 [0.64–0.92]	8.19	22.70
TUG (s)	27	11.80 (3.81)	11.36 (3.72)	0.88 [0.76–0.94]	1.29	3.59
30-s STS (reps)	26	9.77 (3.28)	9.30 (3.26)	0.88 [0.75–0.94]	1.12	3.09

*OLS*  One-leg stand, *TUG*  Timed up and go, *30-s STS*  30-second sit-to-stand, *s*  Seconds, *reps*  Repetitions, *SD*  Standard deviation, *ICC(2,1)* Intraclass correlation coefficient (two-way random effects, single measures, absolute agreement), *CI*  Confidence interval, *SEM*  Standard error of measurement, *SDC95*  Smallest detectable change at 95% confidence

^*^n = 4 (16%) reached the maximum time (60 s)

**Fig. 4:**
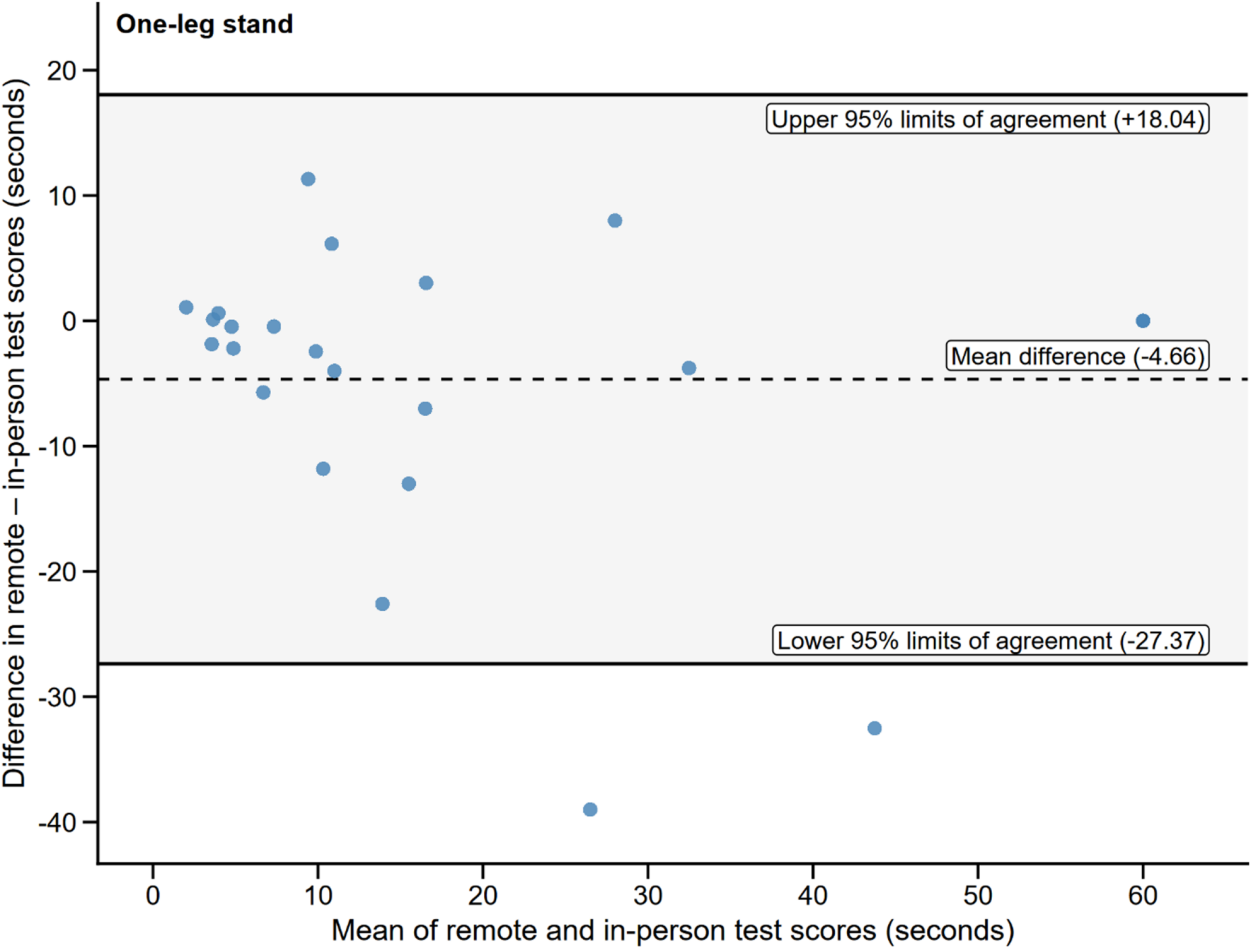
Bland-Altman plot visualising agreement (remote − in person, trial 1) for the one-leg stand (OLS)

**Fig. 5:**
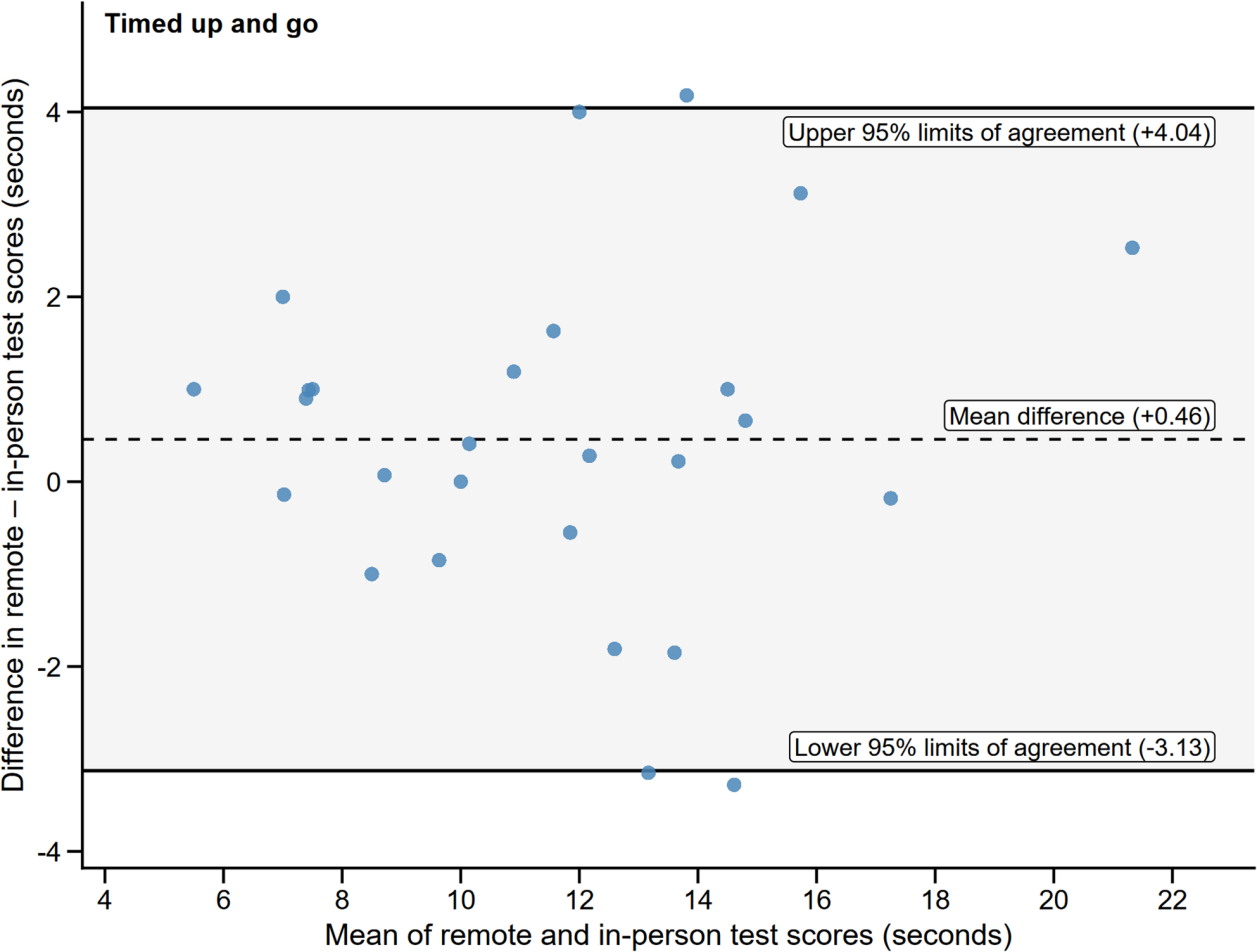
Bland-Altman plot visualising agreement (remote − in person, trial 1) for the Timed Up and Go (TUG)

**Fig. 6:**
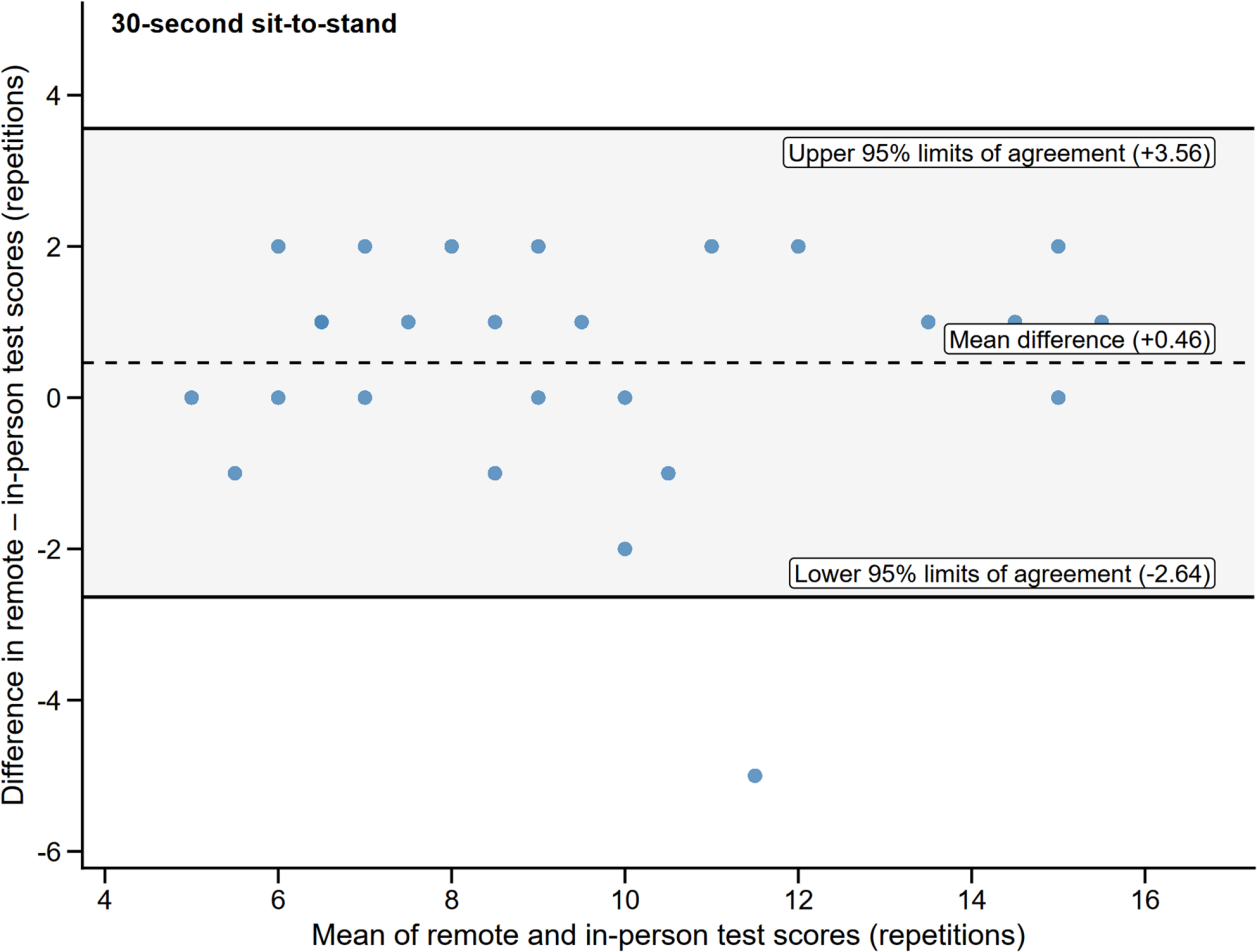
Bland-Altman plot visualising agreement (remote − in person, trial 1) for the 30-second Sit-to-Stand (30-s STS)

The mean difference was − 4.66 s for OLS, 0.46 s for TUG, and 0.46 repetitions for the 30-s STS. Across the Bland–Altman plots (Figs. 4 and 5), visual inspection of the OLS and TUG tests showed that observations with lower mean values clustered more closely around zero. By contrast, larger absolute differences were more common at higher mean values. For the 30-s STS test (Fig. 6), the differences appeared more evenly distributed across the range of mean values.

### Intra- and inter-rater reliability

For intra-rater reliability, ICC ranged from 0.95 to 0.97 in the in-person setting and from 0.80 to 0.98 in the remote setting (Tables 3 and 4). Mean differences between trials were 0.44 s in the in-person setting and 0.29 s in the remote setting for the TUG. For the 30-s STS, mean differences were − 0.50 repetitions in the in-person setting and − 0.15 repetitions in the remote setting. For OLS, the mean difference was 0.49 s in the in-person setting and − 5.82 s in the remote setting. Five participants (19%) reached the maximum OLS time in the in-person setting and four participants (16%) in the remote setting.

**Table 3:** Intra-rater reliability for the in-person assessments

Test	*n*	Trial 1 Mean (SD)	Trial 2 Mean (SD)	ICC(3,1) [95% CI]	Mean difference (95% LoA)	SEM	SDC95
OLS (s)*	27	23.77 (22.24)	23.28 (21.91)	0.97 [0.93–0.99]	0.49 (− 10.13 to 11.11)	3.83	10.62
TUG (s)	28	11.36 (3.65)	10.92 (3.54)	0.95 [0.88–0.98]	0.44 (− 1.76 to 2.64)	0.80	2.20
30-s STS (reps)	26	9.42 (3.20)	9.92 (3.32)	0.96 [0.87–0.99]	−0.50 (− 1.99 to 0.99)	0.54	1.49

*OLS*  One-leg stand, *TUG*  Timed up and go, *30-s STS*  30-second sit-to-stand, *s*  Seconds, *reps*  Repetitions, *SD*  Standard deviation, *ICC(3,1)* Intraclass correlation coefficient (two-way mixed effects, single measures), *CI*  Confidence interval, *LoA*  Limits of agreement, *SEM*  Standard error of measurement, *SDC95*  Smallest detectable change at 95% confidence

^*^n = 5 (19%) reached the maximum test time (60 s)

**Table 4:** Intra-rater reliability for the remote assessments

Test	*n*	Trial 1 Mean (SD)	Trial 2 Mean (SD)	ICC(3,1) [95% CI]	Mean difference (95% LoA)	SEM	SDC95
OLS (s)*	25	18.53 (20.32)	24.35 (22.07)	0.80 [0.57–0.91]	−5.82 (− 30.32 to 18.68)	8.84	24.50
TUG (s)	26	11.72 (3.87)	11.43 (3.52)	0.96 [0.90–0.98]	0.29 (− 1.81 to 2.40)	0.76	2.10
30-s STS (reps)	26	9.77 (3.28)	9.92 (3.40)	0.98 [0.95–0.99]	−0.15 (− 1.59 to 1.28)	0.52	1.43

*OLS*  One-leg stand, *TUG*  Timed up and go, *30-s STS*  30-second sit-to-stand, *s*  Seconds, *reps*  Repetitions, *SD*  Standard deviation, *ICC(3,1)* Intraclass correlation coefficient (two-way mixed effects, single measures), *Mean difference*  Trial 1 − trial 2, *CI*  Confidence interval, *LoA*  Limits of agreement, *SEM*  Standard error of measurement, *SDC95*  Smallest detectable change at 95% confidence

^*^n = 4 (16%) reached the maximum test time (60 s)

For inter-rater reliability, ICC ranged from 0.95 to 0.98 across the three tests (Table 5). Mean differences between the remote and video-recorded assessments were − 0.97 s for OLS, − 0.05 s for TUG, and − 0.16 repetitions for the 30-s STS. Three participants (13.6%) reached the maximum OLS test time (60 s). Technical recording errors precluded analysis of three OLS assessments, reducing the sample size to *n* = 22, below the predefined target of *n* = 25. Limits of agreement, SEM, and SDC95 values for intra- and inter-rater reliability are reported in Tables 3, 4 and 5.

**Table 5:** Inter-rater reliability for the remote assessments and video-recorded assessments

Test	*n*	Remote Mean (SD)	Video-rec. Mean (SD)	ICC(2,1) [95% CI]	Mean difference (95% LoA)	SEM	SDC95
OLS (s)*	22	16.74 (19.23)	17.71 (18.99)	0.98 [0.95–0.99]	−0.97 (− 8.50 to 6.56)	2.72	7.53
TUG (s)	26	11.72 (3.87)	11.76 (4.11)	0.98 [0.96–0.99]	−0.05 (− 1.48 to 1.39)	0.52	1.43
30-s STS (reps)	25	9.84 (3.33)	10.00 (3.32)	0.98 [0.96–0.99]	−0.16 (− 1.38 to 1.06)	0.44	1.22

*OLS*  One-leg stand, *TUG*  Timed up and go, *30-s STS*  30-second sit-to-stand, *s*  Seconds, *reps*  Repetitions, *SD*  Standard deviation, *ICC(2,1)* Intraclass correlation coefficient (two-way random effects, single measures, absolute agreement), *Mean difference*  remote − video recording, *CI*  Confidence interval, *LoA*  Limits of agreement, *SEM*  Standard error of measurement, *SDC95*  Smallest detectable change at 95% confidence

^*^n = 3 (13.6%) reached the maximum test time (60 s)

## Discussion

This study demonstrated sufficient criterion validity and intra- and inter-rater reliability for video-based remote assessment of the OLS, TUG, and 30-s STS in patients with LSS awaiting decompression surgery. ICC estimates for the TUG and 30-s STS were consistently high across analyses, while estimates for the OLS showed greater variability and wider confidence intervals.

### Criterion validity

The ICC estimates for the TUG and 30-s STS (0.88 for both) were comparable to those reported in previous studies examining remote and in-person administration of the same tests in musculoskeletal populations. Dias et al. [44] reported ICC values of 0.82–0.88 for the TUG and 30-s STS in older adults with various musculoskeletal conditions, and Lawford et al. [45] reported ICC values of 0.81–0.82 for these tests in a predominantly older sample with chronic lower-limb musculoskeletal pain. According to the interpretation criteria of Koo and Li [39], both tests demonstrated good-to-excellent criterion validity, with 95% confidence intervals ranging from 0.75 to 0.94. For the OLS (ICC 0.83), the 95% confidence interval ranged from moderate to excellent (0.64–0.92), with the lower bound below the predefined threshold of 0.70, indicating reduced precision. A similar pattern of wider confidence intervals and ceiling effects for single-leg stance has been reported by Lawford et al. [45].

### Intra- and inter-rater reliability

Regarding intra-rater reliability, the in-person assessments showed ICC values ranging from 0.95 to 0.97 across all three tests. These findings are comparable to previous in-person evaluations of the TUG in lumbar degenerative disc disease (ICC = 0.97) [46] and of the 30-s STS in individuals with non-specific low back pain (ICC = 0.94) [21]. For the OLS, the ICC estimate (0.97) was similar to that reported by Maribo et al. [20] (ICC = 0.91), who used the same “worst leg” scoring approach, although the test was performed with eyes closed and with a 30-second time ceiling in patients with low back pain. According to Koo and Li [39], the corresponding 95% confidence intervals for the TUG and 30-s STS ranged from 0.87 to 0.99, indicating good-to-excellent intra-rater reliability, and were excellent for the OLS (0.93–0.99).

For the remote assessments, ICC estimates were 0.96 for the TUG and 0.98 for the 30-s STS, consistent with Lawford et al. [45], who reported high test–retest reliability for both tests when administered via telehealth one week apart (ICC 0.89–0.94). Based on the 95% confidence intervals, both the TUG and 30-s STS (0.90–0.99) demonstrated excellent intra-rater reliability [39]. For OLS, the ICC estimate indicated sufficient reliability (ICC = 0.80), but the confidence interval was wide, spanning the moderate-to-excellent range (0.57–0.91) according to Koo and Li [39], reflecting reduced precision compared with the TUG and 30-s STS. Comparable variability in ICC values for single-leg balance tests assessed via telehealth was also reported by Lawford et al. [45].

For inter-rater reliability, based on independent rescoring of the video-recorded remote assessments, ICC estimates were 0.98 (95% CI 0.96–0.99) for both the TUG and 30-s STS, and 0.98 (95% CI 0.95–0.99) for the OLS. According to Koo and Li [39], these values indicate excellent reliability. These findings align with Dias et al. [44], who evaluated inter-rater reliability for the same tests during live remote assessment, in which two raters simultaneously and independently scored performance via video in older adults with mixed musculoskeletal conditions, reporting ICC values ranging from 0.97 to 0.99. For the OLS, the inter-rater sample size was reduced to *n* = 22 due to technical recording errors. Despite the smaller sample, the confidence intervals remained within the excellent range (0.95–0.99). The narrow confidence intervals indicate high precision in the inter-rater agreement estimates.

### Measurement error parameters

Although the ICC analyses showed sufficient reliability and criterion validity across tests, the limits of agreement, SEM, and SDC95 were relatively large compared with the mean test scores and should be considered when interpreting individual-level change. For example, the SDC95 of 3.59 s for the TUG when comparing remote and in-person assessments indicates that a patient would need to improve or deteriorate by more than this amount for the change to be considered beyond measurement error [28]. Given that the mean TUG scores ranged from 11.36 to 11.8 s, an SDC95 of 3.59 s represents a substantial proportion of the mean values.

However, the precision of these measurement error estimates is limited, as the sample size was determined to ensure adequate precision for ICC analyses rather than to estimate measurement error parameters [28, 47]. Larger samples are needed to obtain more precise estimates of these parameters [47] and to determine whether measurement error parameters vary across the range of mean test values, as suggested by inspection of the Bland–Altman plots [48]. In addition, the responsiveness of these physical capacity tests when administered remotely in patients with LSS remains largely unexplored and warrants further investigation.

### Potential factors influencing between-setting variability

Across all three tests, ICC values for between-setting comparisons (criterion validity) were lower than those for within-setting reliability. This pattern is expected, as assessments conducted across different environments introduce additional sources of measurement error [28]. In the present study, this may reflect challenges in standardising home-based testing equipment and environments, despite efforts to align conditions across settings (e.g., chair height, walking distance, and a clear, level, firm floor surface). Similar challenges have been reported in previous research comparing remote and in-person administration of these tests in older adults [49].

For OLS specifically, the lower ICC estimates compared with the TUG and 30-s STS may partly reflect the inherent measurement variability in single-leg balance performance [50]. Furthermore, OLS performance improved notably between the two remote trials (mean difference of − 5.82 s), whereas it remained stable in the in-person setting (mean difference 0.49 s). This remote-specific variability might have increased error variance, thereby reducing the between-setting ICC [28]. This pattern could indicate familiarisation effects unique to remote OLS testing, as previously documented in repeated remote balance assessments in older adults [49, 51]. These findings suggest that familiarisation attempts may be warranted before remote OLS assessment in clinical practice and should be further evaluated in future research. In contrast, the stable performance observed for the TUG and 30-s STS across repeated remote trials suggests that familiarisation may be less important for these tests.

### Generalisability

Our sample was similar to data from the Swedish National Spine Register (SweSpine) for surgically treated patients with central LSS [31] in terms of age (69 vs. 68 years) and the proportion experiencing leg pain for longer than 1 year (61% vs. 65%). However, our sample showed higher rates of previous spine surgery (32% vs. 21%) but lower self-reported disability (ODI 36 vs. 43).

Compared with two recent SweSpine cohort studies, our sample had a similar BMI (28.1 vs. 27.7) [52] but a markedly higher proportion of university-educated individuals (50% vs. 28%) [53]. As a result, while the sample aligns with patients undergoing LSS surgery in Sweden across several key demographics, it appears to be a somewhat more educated and less disabled subgroup. Taken together, these characteristics and the requirement to use electronic identification to access the videoconferencing platform, suggest that the findings may be less generalisable to patients with lower educational attainment, lower digital literacy, limited access to smart devices, or greater preoperative disability.

### Methodological strengths

A key strength of this study was its integrated design, which enabled simultaneous evaluation of remote and in-person assessments, intra-rater reliability in both settings, and inter-rater reliability through independent video rescoring of remote test sessions. Another strength was assessing participants in their home environments, unlike previous studies that used controlled laboratory settings [54–56]. This provided a more realistic evaluation context and potentially enhanced the external validity of our findings. The results are consistent with those of previous studies using similar home-based setups [44, 45].

Additional strengths include the a priori sample size calculation to ensure adequate statistical precision, the randomised order of test settings to reduce test-order bias, and careful characterisation of the study population. Furthermore, safety outcomes were proactively monitored and reported, consistent with recent recommendations for telehealth-rehabilitation research [57].

### Methodological limitations

This study was limited by single-site recruitment, which may reduce its generalisability. Additionally, inter-rater reliability was assessed by an independent rater who re-scored video recordings of the remote assessments. Although this method evaluates scoring consistency, it does not capture the full range of inter-rater reliability [28], including potential variability in how different raters conduct tests in real time. Future studies should include a live second rater to provide a more comprehensive evaluation of inter-rater reliability in the remote setting.

Another limitation is that the measurement properties of the OLS may have been compromised by our scoring strategy, which used a single OLS attempt of the patient’s “worst leg” [20]. This method was chosen for its clinical relevance, as balance assessments in practice typically focus on the most impaired side. However, relying on a single attempt likely increased random error compared with averaging multiple attempts. Averaging repeated measurements is well known to reduce random error and improve the stability of results [28, 41]. Future research should consider averaging multiple attempts to obtain a more reliable estimate of balance performance. Additionally, larger studies are needed to determine the accuracy of reliability estimates and the extent of detectable change at the individual level.

### Clinical implications

Remote testing via video facilitates hybrid care pathways (e.g., an initial in-person evaluation followed by remote follow-up), a model increasingly promoted in low back pain rehabilitation [58, 59]. The TUG and 30-s STS showed particularly strong results, supporting their use as reliable complements to in-person assessments in patients with LSS. Although the OLS met the predefined ICC threshold for adequate criterion validity, its greater uncertainty, more frequent ceiling effects, and higher relative measurement error than the TUG and 30-s STS suggest cautious interpretation at the individual level.

No adverse events were observed during any assessments, indicating that, with appropriate patient instructions and safety measures, physical capacity testing can be conducted safely at home and evaluated remotely via video call for patients with LSS awaiting surgery. These findings align with previous studies reporting the safety and feasibility of video-based assessments in older adults [49, 60]. Nonetheless, careful patient selection and preparation remain essential, and individuals at high risk of falls should either be excluded from remote testing or be assessed with a family member or caregiver present for support [51, 57].

## Conclusions

The findings of this study indicate that remote video assessment of the OLS, TUG, and 30-s STS may serve as a valid and reliable complement to in-person evaluation for patients with LSS scheduled for decompression surgery. However, the greater measurement variability observed for the OLS warrants caution when interpreting remote OLS results. Overall, the results support blended care models that combine in-person and remote assessments, aligning with the global shift towards e-health. Further research is needed to examine measurement error and responsiveness for these tests and to determine whether alternative OLS scoring methods or familiarisation attempts before remote testing could improve the robustness of OLS results.

## Data Availability

The datasets used and analysed in this study are available from the corresponding author upon reasonable request.

## Abbreviations

LSS: Lumbar spinal stenosis
OLS: One-leg stand
TUG: Timed up and go
30-s STS: 30-second sit-to-stand
BMI: Body mass index
ODI: Oswestry Disability Index 2.0
NPRS: Numerical Pain Rating Scale
